# Effects of renal impairment and hemodialysis on the pharmacokinetics and safety of HRS-8427, a siderophore cephalosporin for Gram‐negative bacterial infections

**DOI:** 10.1128/aac.01095-25

**Published:** 2026-01-13

**Authors:** Yuanhao Wu, Qiwen Han, Haijing Yang, Jun Xue, Lili Wang, Xiaomeng Mao, Tengrui Yin, Hao Jiang, Sheng Xu, Yuanyuan Huang, Ting Wang, Yan He, Yuanyuan Luo, Wenli Chen, Jing Zhang

**Affiliations:** 1 Huashan Hospital Affiliated to Fudan University, Shanghai, China; 2 Jiangsu Hengrui Pharmaceuticals Co., Ltd., Shanghai, China; 3 The First Hospital of Lanzhou University, Lanzhou, China; 4 The Affiliated Hospital of Guizhou Medical University 74720, Guiyang, China; 5 The Affiliated Hospital of Xuzhou Medical University 117910, Xuzhou, China; 6 The Central Hospital of Wuhan 577528, Wuhan, China; University of Houston, Houston, Texas, USA

**Keywords:** siderophore cephalosporin, pharmacokinetics, renal impairment, hemodialysis

## Abstract

HRS-8427, a development-stage siderophore cephalosporin antibiotic, is being investigated for the treatment of aerobic Gram-negative bacterial infections, encompassing urinary tract infections and pulmonary infections. Participants with these indications frequently exhibit concomitant renal impairment (RI). Data from clinical studies suggest that ~60% to ~70% of unchanged HRS-8427 is excreted renally. A phase 1, multicenter, open-label study evaluated the effects of RI on the pharmacokinetics and safety of HRS-8427. In sub-study 1, 21 participants with mild to severe RI who were not on dialysis and six participants with normal renal function received single doses of 1,000 mg HRS-8427. In sub-study 2, six participants with end-stage renal disease (ESRD) requiring hemodialysis (HD) received single doses of the 1,000 mg HRS-8427 under dialysis and non-dialysis conditions, respectively. Plasma HRS-8427 area under the concentration-time curve from zero to infinity (AUC_0–∞_) was ~1.2-fold, ~1.4-fold, 2.0-fold, and ~2.0-fold higher, respectively, in participants with mild, moderate, severe RI, and ESRD (without HD) relative to healthy controls. In dialysis-dependent subjects, the systemic exposure of HRS-8427 when dosed before HD was equivalent to 50.6% of that when dosed after HD. Adverse events (AEs) were mostly mild, and RI did not appear to be associated with an increased risk of AEs.

**CLINICAL TRIALS:**

This study is registered with http://www.chinadrugtrials.org.cn/ as CTR20230658.

## INTRODUCTION

Antimicrobial resistance represents an escalating global health emergency, currently accounting for approximately 700,000 annual deaths worldwide. Projections indicate this toll could surge to 10 million deaths annually by 2050 (1). The alarming rise of β-lactamase-mediated resistance in Gram-negative bacteria jeopardizes the efficacy of β-lactams—the most extensively used antibiotic class in clinical medicine (2). HRS8427, a novel siderophore cephalosporin antibiotic in late-stage clinical development, demonstrates potent *in vitro* activity against a broad spectrum of multidrug-resistant Gram-negative bacteria, including Enterobacteriaceae, *Pseudomonas aeruginosa*, and *Acinetobacter baumannii*. HRS-8427 is currently undergoing clinical investigations for the treatment of adult bacterial pneumonia (a phase 2 study, ClinicalTrials.gov identifier NCT06841731) and adult complicated urinary tract infections (a phase 2 study, ClinicalTrials.gov identifier NCT06144060; and a phase 3 study, ClinicalTrials.gov identifier NCT06569056). Among similar products, cefiderocol (Fetroja) is currently the only approved siderophore antibiotic for clinical use, although it has not yet been marketed in China (3). The successful development of HRS-8427 may provide more treatment options for infections resistant to β-lactam antibiotics caused by Gram-negative bacteria.

The safety, tolerability, and pharmacokinetics (PK) of HRS-8427 have been evaluated in a single- and multiple-dose phase 1 study (unpublished data on file). HRS-8427 exhibited essentially linear PK characteristics over the dose range of 400 to 6,000 mg. In healthy participants, the terminal elimination half-life (*t*_1/2_) of HRS-8427 was 3.9 to 4.3 h. The primary clearance pathway of HRS-8427 involves urinary excretion, with 62% to 71% of the administered dose recovered in urine as unchanged parent drug. This finding indicates that renal clearance is a major route of elimination.

Patients with chronic kidney disease (CKD) and end-stage renal disease (ESRD) exhibit compromised humoral and cellular immunity, which results in a heightened susceptibility to infections (4) and thus positions them as potential candidates for HRS-8427 therapy. Nevertheless, renal impairment (RI) can alter the metabolism or transport of drugs for which the kidney is the primary route of excretion. Consequently, characterizing the PKs of HRS-8427 in participants with RI is essential to establish rational dosing regimens for aerobic Gram-negative bacteria infections in this population. This study aimed to evaluate the PKs, safety, and tolerability of HRS-8427 in clinically stable participants with mild, moderate, and severe RI, as well as in those undergoing hemodialysis (HD). Additionally, the effect of HD on the clearance of HRS-8427 was also investigated.

## RESULTS

### Participants

A total of 33 participants were enrolled and completed the study. Demographic and baseline characteristics are summarized in Table 1. Participants had a mean age of 47.1 years (range, 26 to 70 years) and a mean body mass index (BMI) of 25.1 kg/m^2^ (range, 18.3 to 32.3 kg/m^2^). All participants enrolled in this study were of Chinese ethnicity, and 25 of the participants were male. Demographic characteristics of participants with normal renal function were generally comparable to those of the patients in the RI groups.

**TABLE 1:** Demographic and baseline characteristics^*a*^

Characteristic (unit)	Renal function group				
	Normal (*n* = 6)	Mild (*n* = 7)	Moderate (*n* = 8)	Severe (*n* = 6)	ESRD (*n* = 6)
Gender, *n* (%)					
Male	5 (83.3)	4 (57.1)	7 (87.5)	4 (66.7)	5 (83.3)
Female	1 (16.7)	3 (42.9)	1 (12.5)	2 (33.3)	1 (16.7)
Ethnicity, *n* (%)					
Chinese	6 (100)	7 (100)	8 (100)	6 (100)	6 (100)
Caucasian	0	0	0	0	0
Mean age, years (range)	47.3 (38–57)	46 (26–70)	45 (30–65)	54.5 (32–68)	43.7 (36–59)
Mean weight, kg (range)	70.92 (63.6–78.9)	72.11 (49.4–93.1)	77.09 (59.0–92.2)	67.4 (49.6–89.4)	64.7 (53.0–84.6)
Mean BMI, kg/m² (range)	24.9 (23.1–26.0)	25.9 (21.8–28.9)	26.8 (22.2–32.3)	24.6 (20.4–31.1)	22.7 (18.3–28.3)
Mean eGFR, mL/min (range)	102.0 (94.4–113.6)	72.5 (59.9–83.8)	43.2 (30.3–50.2)	21.4 (15.9–29.0)	5.3 (3.7–7.9)

^*a*^ BMI, body mass index; eGFR, estimated glomerular filtration rate; ESRD, end‐stage renal disease.

### Pharmacokinetics assessments

#### Effect of RI

**Fig 1:**
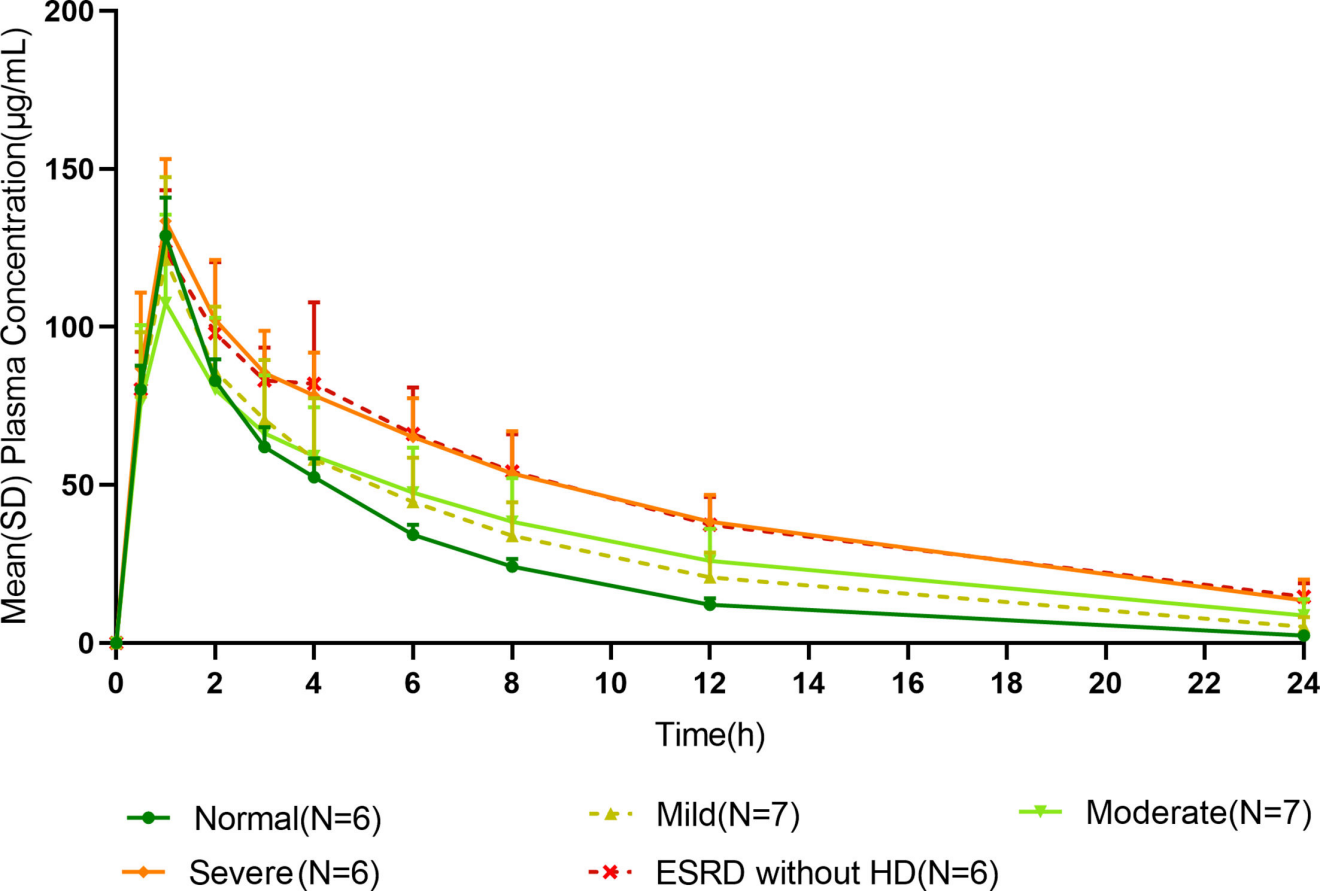
Mean (SD) plasma HRS-8427 concentration profiles following a single intravenous infusion of 1,000 mg HRS-8427 over 1 h. SD, standard deviation; ESRD, end‐stage renal disease; HD, hemodialysis.

The time course of mean plasma HRS-8427 concentration profiles following a single intravenous infusion 1,000 mg over 1 h in each renal function group is shown in Fig. 1. Peak plasma concentrations of HRS-8427 were achieved immediately after completion of the infusion. A remarkable alteration in plasma concentration profiles was observed, which was directly correlated with the severity of renal function. The summary of the PK parameters of HRS-8427 is presented in Table 2. The area under the plasma concentration-time curve from time 0 to the last quantifiable concentration (AUC_0–t_), area under the plasma concentration-time curve from time 0 to extrapolated infinity (AUC_0_**_–∞_**), and *t*_1/2_ of HRS-8427 demonstrated progressive increases with escalating degrees of RI. The geometric mean *t*_1/2_ values in normal, mild, moderate, severe, and ESRD (without HD) groups were 4.7, 5.6, 7.1, 7.6, and 9.1 h, respectively. The comparisons of PK parameters between RI groups and the normal group are presented in Table 3. Compared with the normal group, the geometric mean ratios and 90% confidence intervals (CIs) of total exposure (AUC_0–∞_) were 1.2 (0.9, 1.7), 1.4 (1.0, 2.0), 2.0 (1.4, 2.8), and 2.0 (1.4, 2.8) for participants with mild, moderate, and severe RI, as well as those with ESRD without HD, respectively, indicating that HRS-8427 exposure increased among participants with deteriorating renal function. The geometric mean ratios of CL relative to those in the normal renal function group were 0.81, 0.71, 0.50, and 0.51, respectively. A similar trend was observed for renal clearance (CL_*R*_), reflecting the compromised renal excretory capacity across impairment grades. Geometric mean values of volume of distribution (*V*_*z*_) and plasma peak concentration (*C*_max_) remained relatively consistent across all renal function groups.

**TABLE 2:** PK parameters of HRS-8427 stratified by group^*b*^

PK parameter	Renal function group					
	Normal (*N* = 6)	Mild (*N* = 7)	Moderate (*N* = 7)	Severe (*N*=6)	ESRD (without HD, *N* = 6)	ESRD (with HD, *N* = 6)
*C*_max_ (μg/mL)	128 (9.7)	119 (21.6)	104 (27.6)	132 (15.0)	119 (16.1)	105 (24.0)
*T*_max_ (h)	1.03 (1.02–1.03)	1.00 (1.00–1.03)	1.02 (0.983–1.12)	1.00 (0.967–1.00)	1.02 (0.950–1.08)	0.967 (0.933–1.03)
AUC_0–t_ (h·μg/mL)	590 (8.5)	709 (29.2)	814 (35.8)	1,150 (29.3)	1,160 (22.2)	541 (20.8)
AUC_0–∞_ (h·μg/mL)	607 (9.0)	753 (30.6)	857 (35.6)	1,220 (26.4)	1,180 (22.3)	603 (18.3)
*t*_1/2_ (h)	4.74 (12.1)	5.56 (30.5)	7.12 (27.9)	7.59 (23.4)	9.11 (12.6)	8.87 (10.8)
*V*_*z*_ (L)	11.3 (9.7)	10.7 (33)	12 (25.6)	9.01 (14.4)	11.1 (12.2)	21.2 (14.5)
CL (L/h)	1.65 (9.0)	1.33 (30.6)	1.17 (35.6)	0.823 (26.4)	0.844 (22.3)	1.66 (18.3)
CL_*R*_ (L/h)	0.935 (10.4)	0.751 (30.9)	0.452 (36.6)	0.250 (26.6)	0.00632 (88.9)	0.001 (191.6)
fe%	56.7 (9.0)	56.5 (11.37)	38.7 (39.3)	30.4 (10.6)	0.642 (98.9)^*a*^	0.0557 (223)^*a*^
CL_*D*_ (L/h)	NA	NA	NA	NA	NA	2.75 (24.2)
Ar	NA	NA	NA	NA	NA	463 (18.4)

^*a*^ The parameter derived from urine concentrations of HRS-8427 is presented for the three ESRD participants capable of producing urine samples.

^*b*^ Values are presented as geometric mean (geometric mean CV%), except for *T*_max_, which is shown as median (minimum ~ maximum). ESRD, end‐stage renal disease; *C*_max_, maximum observed plasma concentration; *T*_max_, time to *C*_max_; AUC_0–t_, area under the plasma concentration-time curve from time 0 to the last measurable time point; AUC_0–∞_, area under the plasma concentration-time curve from time 0 to infinity; *t*_1/2_, terminal elimination half-life; *V*_*z*_, volume of distribution during terminal elimination phase; CL, total drug clearance from plasma; CL_*R*_, renal clearance of drug; fe, fraction of dose excreted unchanged into urine; CL_*D*_, hemodialysis clearance; Ar, cumulative unchanged drug recovery in dialysate. NA, not available.

**TABLE 3:** Comparisons of PK parameters of HRS-8427 between RI groups and normal renal function group^*a*^

PK parameter	Ratio of geometric mean (90% CI)			
	Mild vs normal	Moderate vs normal	Severe vs normal	ESRD without HD vs normal
*C*_max_	0.927 (0.715–1.204)	0.813 (0.626–1.056)	1.031 (0.786–1.351)	0.926 (0.697–1.230)
AUC_0–t_	1.202 (0.861–1.680)	1.380 (0.988–1.928)	1.956 (1.383–2.768)	1.967 (1.367–2.830)
AUC_0–∞_	1.241 (0.894–1.723)	1.413 (1.018–1.962)	2.003 (1.425–2.815)	1.952 (1.366–2.789)
*t*_1/2_	1.175 (0.888–1.553)	1.504 (1.138–1.989)	1.603 (1.199–2.142)	1.925 (1.420–2.609)
CL	0.806 (0.580–1.119)	0.707 (0.509–0.982)	0.499 (0.355–0.702)	0.513 (0.358–0.732)
*V*_*Z*_	0.947 (0.725–1.236)	1.065 (0.815–1.390)	0.800 (0.607–1.055)	0.986 (0.738–1.318)
CL_*R*_	0.803 (0.428–1.509)	0.483 (0.257–0.908)	0.267 (0.139–0.514)	0.007 (0.003–0.015)

^*a*^ ESRD, end‐stage renal disease; *C*_max_, maximum observed plasma concentration; AUC_0–t_, area under the plasma concentration-time curve from time 0 to the last measurable time point; AUC_0–∞_, area under the plasma concentration-time curve from time 0 to infinity; *t*_1/2_, terminal elimination half-life; CL, total drug clearance from plasma; *V*_*Z*_, volume of distribution during terminal elimination phase; CL_*R*_, renal clearance of drug.

**Fig 2:**
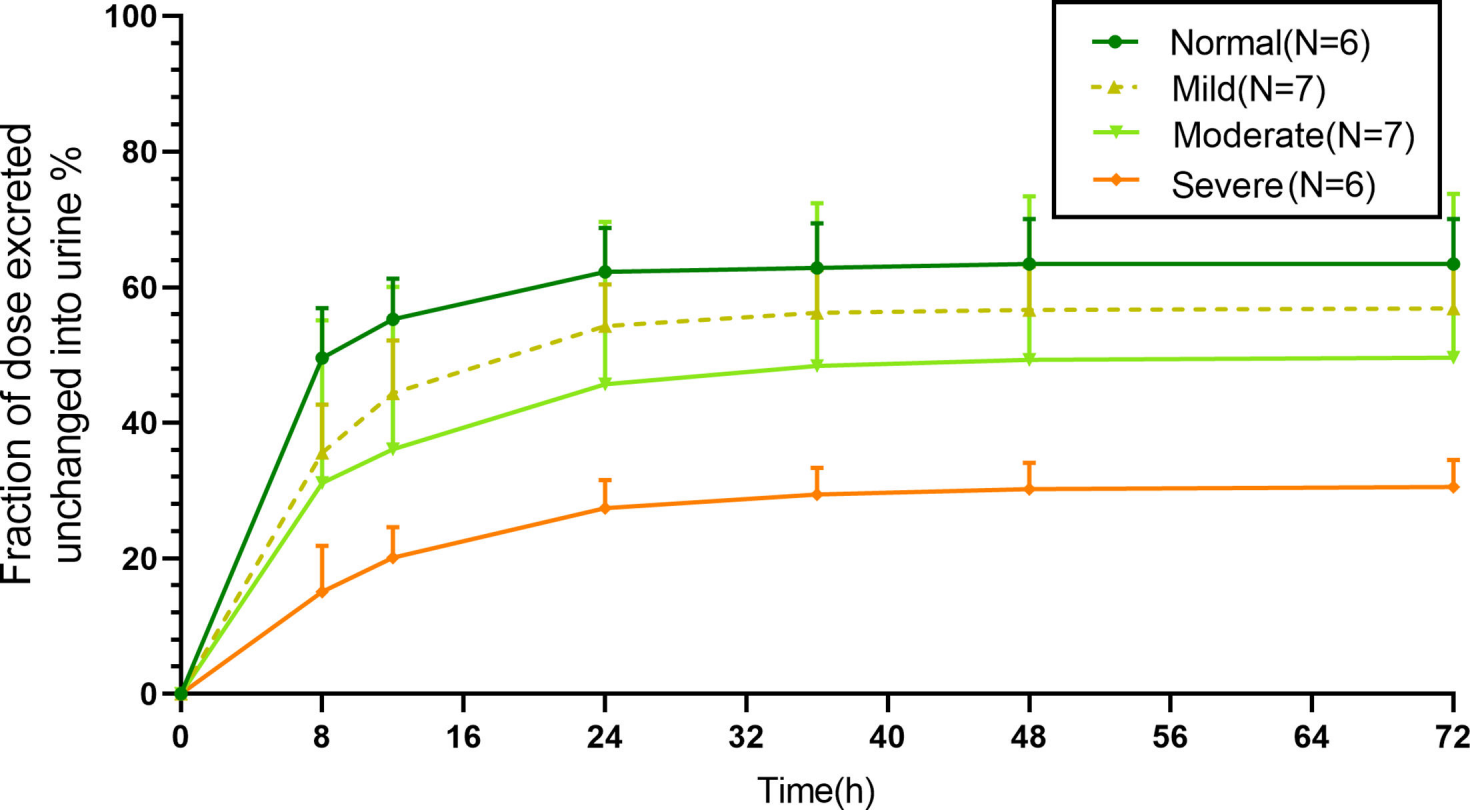
Mean (SD) fraction of HRS-8427 dose excreted in urine following a single intravenous infusion of 1,000 mg of HRS-8427 over 1 h.

The cumulative urinary excretion of unchanged HRS-8427 across different grades of RI is shown in Fig. 2. In all participants, urinary excretion of the unchanged drug was predominantly completed within 24 h post-dose, thereby confirming the adequacy of the urine collection period for participants with RI. As detailed in Table 4, the mean urinary recovery (fe%) of unchanged HRS-8427 in participants with mild RI was 56.5% of the administered dose—comparable to values observed in the healthy control group. This recovery rate progressively declined to 38.7% and 30.4% in participants with moderate and severe RI, respectively. In ESRD participants (without HD), who were essentially anuric, urinary excretion of unchanged HRS-8427 accounted for only 0.6% of the dose.

**TABLE 4:** Treatment-emergent adverse events (TEAEs)^*a*^

System organ class and preferred term	Number (%) of participants with renal function						
	Normal (*n* = 6)	Mild (*n* = 7)	Moderate (*n* = 8)	Severe (*n* = 6)	ESRD without HD (*n* = 6)	ESRD with HD (*n* = 6)	Total (*n* = 33)
Number of participants with at least one adverse event	1 (16.7)	3 (42.9)	3 (37.5)	2 (33.3)	0 (0.0)	0 (0.0)	9 (27.3)
Musculoskeletal and connective tissue disorders	0 (0.0)	0 (0.0)	0 (0.0)	1 (16.7)	0 (0.0)	0 (0.0)	1 (3.0)
Arthritis	0 (0.0)	0 (0.0)	0 (0.0)	1 (16.7)	0 (0.0)	0 (0.0)	1 (3.0)
Investigations	0 (0.0)	2 (28.6)	2 (25.0)	1 (16.7)	0 (0.0)	0 (0.0)	5 (15.2)
Neutrophil percentage decreased	0 (0.0)	0 (0.0)	1 (12.5)	0 (0.0)	0 (0.0)	0 (0.0)	1 (3.0)
Neutrophil count decreased	0 (0.0)	0 (0.0)	1 (12.5)	0 (0.0)	0 (0.0)	0(0.0)	1(3.0)
Urinary occult blood positive	0 (0.0)	1 (14.3)	0 (0.0)	0 (0.0)	0 (0.0)	0 (0.0)	1 (3.0)
White blood cells urine positive	0 (0.0)	1 (14.3)	0 (0.0)	1 (16.7)	0 (0.0)	0 (0.0)	2 (6.1)
Red blood cells urine positive	0 (0.0)	1 (14.3)	0 (0.0)	0 (0.0)	0 (0.0)	0 (0.0)	1 (3.0)
Protein urine present	0 (0.0)	1 (14.3)	0 (0.0)	0 (0.0)	0(0.0)	0 (0.0)	1 (3.0)
White blood cell count decreased	0 (0.0)	0(0.0)	1(12.5)	0(0.0)	0 (0.0)	0 (0.0)	1 (3.0)
Bile acids increased	0 (0.0)	0 (0.0)	1 (12.5)	0 (0.0)	0 (0.0)	0 (0.0)	1 (3.0)
Infections and infestations	0 (0.0)	1 (14.3)	1 (12.5)	0 (0.0)	0 (0.0)	0 (0.0)	2 (6.1)
Upper respiratory tract infection	0 (0.0)	1 (14.3)	1 (12.5)	0 (0.0)	0 (0.0)	0 (0.0)	2 (6.1)
Skin and subcutaneous tissue disorders	1 (16.7)	0 (0.0)	0 (0.0)	0 (0.0)	0 (0.0)	0 (0.0)	1 (3.0)
Drug eruption	1 (16.7)	0 (0.0)	0 (0.0)	0 (0.0)	0 (0.0)	0 (0.0)	1 (3.0)
Blood and lymphatic system disorders	0 (0.0)	0 (0.0)	1 (12.5)	0 (0.0)	0 (0.0)	0 (0.0)	1 (3.0)
Anemia	0 (0.0)	0 (0.0)	1 (12.5)	0 (0.0)	0 (0.0)	0 (0.0)	1 (3.0)

^*a*^ ESRD, end‐stage renal disease; HD, hemodialysis.

#### Effect of HD

**Fig 3:**
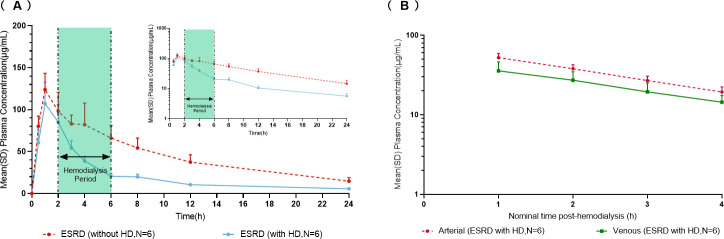
(**A**) Mean (SD) plasma HRS-8427 concentration profiles without HD (dosing post HD) and with HD (dosing prior to HD) following single intravenous infusion of 1,000 mg of HRS-8427 over 1 h (insert: semilogarithmic scale). (**B**) Mean plasma HRS-8427 concentration profiles of the arterial and venous after HD following a single intravenous infusion of 1,000 mg of HRS-8427 over 1 hour (semilogarithmic scale). ESRD, end‐stage renal disease; HD, hemodialysis.

The mean plasma concentration profiles of HRS-8427 following a single 1,000 mg intravenous infusion over 1 h were compared between ESRD participants with and without HD (Fig. 3A). HD initiated after drug administration resulted in rapid elimination of HRS-8427. During an approximate 4-hour HD period, venous HRS-8427 concentrations remained lower than those in the off-dialysis state. Furthermore, the arterio-venous kinetics of HRS-8427 plasma concentrations in HD-treated ESRD participants exhibited parallel trends on semi-logarithmic scales (Fig. 3B), indicating a constant arterio-venous concentration ratio and consistent extraction efficiency throughout HD session. The geometric mean cumulative unchanged drug recovery in dialysate (Ar) was 463 mg (95% CI, 383 to 560), indicating that approximately half of the administered dose of HRS-8427 was removed during a 4-hour HD session.

### Safety

HRS-8427 was generally well tolerated in the study participants. No deaths or serious adverse events (AEs) were reported during the study. No participant discontinued the study drug or withdrew prematurely due to AEs. Table 4 summarizes the treatment-emergent adverse events (TEAEs) by system organ class. For the full study population, nine subjects (27.3%) reported TEAEs during the conduct of the study. The most frequently reported TEAEs were positive urine white blood cells and upper respiratory tract infection. All these events were assessed as unrelated to the study drug.

Three participants experienced drug-related TEAEs, which included drug eruption, urinary occult blood positive, and increased bile acids for participants in the normal, mild, and moderate groups, respectively. With the exception of the moderate-severity drug eruption, all other TEAEs were mild. There was no evidence of an increased incidence or severity of AEs with decreased renal function. No clinically significant trends or shifts from baseline were reported for physical examination, vital signs, or electrocardiographic findings.

## DISCUSSION

HRS-8427, a next-generation siderophore cephalosporin antibiotic, exhibits a dual-mechanism action profile. Firstly, by using the iron transport system analogous to the “Trojan horse” strategy to enter Gram-negative bacteria (5), it chelates extracellular ferric iron to form a complex that hijacks bacterial iron transport proteins, enabling active translocation across the outer cell membrane and accumulation in the periplasmic space at elevated concentrations. Upon entering the bacterial cytoplasm, it binds to penicillin-binding proteins, potently inhibiting cell wall biosynthesis and exerting both bacteriostatic and bactericidal effects (6). Secondly, in contrast to many cephalosporins and carbapenems, HRS-8427 demonstrates intrinsic resistance to β-lactamase-mediated hydrolysis, ensuring enhanced stability (7). Therefore, this antibiotic displays potent antibacterial potential against Gram-negative aerobic bacteria.

The first-in-human study of HRS-8427 demonstrated linear PK characteristics across the dose range of 400 to 6,000 mg in healthy participants and showed an overall favorable safety and tolerability profile. Leveraging *in vitro* pharmacodynamic findings and referencing the clinical dosing regimen of the analogous siderophore antibiotic cefiderocol, the clinically effective dose range of HRS-8427 was estimated to be 1,000 to 2,000 mg. Population PK modeling further revealed that ESRD participants exhibited approximately 2.1-fold higher HRS-8427 exposure than participants with normal renal function. Collectively, these data supported that the selection of a single 1,000 mg dose for this RI study could ensure an adequate safety margin.

The glomerular filtration rate (GFR) is widely regarded as the optimal comprehensive screening tool for evaluating renal function. The modification of diet in renal disease (MDRD) and Chronic Kidney Disease Epidemiology Collaboration equations are two prominent formulas for calculating estimated GFR (eGFR) in adults. The MDRD method has seen increasing integration into approved new drug labeling for dosing recommendations (8). The abbreviated MDRD equation, which includes only plasma creatinine, gender, age, and ethnicity, has been the most widely used for GFR assessment in Western populations (9). Race emerges as a critical factor influencing the accuracy of the MDRD equation. A Chinese-modified MDRD equation was developed using data from 684 Chinese participants with CKD, demonstrating significant advantages across different CKD stages and proving suitable for clinical application. However, the equation exhibited limited generalizability to non-White and non-Black populations and has not been validated in large-scale diverse cohorts (10). Given that all participants planned to be enrolled in this study were Chinese adults, this formula was more appropriate for renal function classification.

HRS-8427 exposure increased in participants with moderate and severe RI and ESRD compared to normal renal function controls. The results suggest that rational dosing adjustments are warranted for these patient populations to maintain drug exposures comparable to those in healthy controls. Despite the observed increase in systemic exposure (approximately 2-fold) in participants with severe RI and ESRD, HRS-8427 was well-tolerated in this single-dose study, with no apparent increase in the incidence or severity of AEs compared to participants with normal renal function. This favorable safety profile at higher exposures naturally raises the question of whether dose adjustment is strictly necessary. However, the recommendation for dose reduction is based on the principle of maintaining drug exposure within the range established for patients with normal renal function in pivotal clinical trials. This approach aims to minimize potential unknown risks associated with sustained higher exposure, particularly in these vulnerable populations who often have multiple comorbidities and may receive multiple courses of therapy. Whereas the single-dose data are reassuring, the long-term safety of sustained double-fold exposure has not been evaluated. This conservative strategy aligns with regulatory guidance for renally cleared drugs and is consistent with the approach taken for other antibiotics in this class, such as cefiderocol. Therefore, while the current safety data are encouraging, dose adjustment remains the recommended strategy to ensure an optimal benefit-risk profile until further clinical experience with multiple doses in the target population is available. In dialysis-dependent subjects, the systemic exposures of HRS-8427 dosing before HD were equivalent to 50.6% of that when dosing after HD. Therefore, HD-dependent participants are recommended to receive the drug after completing a HD session to avoid compromising drug effect.

In this study, one healthy participant experienced a drug eruption AE. Approximately 33 h after drug administration, the participant developed red, maculopapular rashes on the trunk (chest and abdomen) and limbs, accompanied by pruritus. Results of routine blood tests and total immunoglobulin E (IgE) assay were within normal ranges, and vital signs showed no abnormalities. This participant recovered 21 days after concomitant medication administration. The drug eruption AE observed in this study differed from the immediate type I allergic reactions reported in the first human study of HRS-8427, where three participants experienced reactions within 30 min of infusion initiation. These symptoms manifested as urticarial rashes with pruritus across multiple body sites, resolving within 2 h after drug discontinuation, and one participant also experienced mild chest tightness. Given the distinct clinical presentations and timelines, the observed drug eruption AE in this study may not represent an allergic reaction to HRS-8427. Supported by findings from the cefiderocol RI study, one participant with moderate RI reported a drug eruption, and another experienced a urticaria AE. Analysis of blood samples failed to detect IgE and immunoglobulin G antibodies against cefiderocol (11).

Urinary leukocyte positivity was observed in one participant with mild RI and one with severe RI. Given that prior studies have reported urinary leukocyte positivity on microscopic urinalysis in participants with impaired renal function, these two cases are likely related to changes associated with the participants’ underlying renal disease status (12, 13). Despite limited sample sizes across renal function groups, no statistically significant or clinically relevant trends in AEs were observed among different groups, even as HRS-8427 systemic exposure increased progressively with worsening RI.

## MATERIALS AND METHODS

### Study design

This was a phase 1, non-randomized, multicenter, open-label study. The primary objective of the study was to compare the PKs of HRS-8427 in adult participants with mild, moderate, or severe RI, and ESRD on HD, to those in matched healthy adult participants. Secondary objectives were to evaluate the safety and tolerability of a single dose of HRS-8427 and to determine the proportion of HRS-8427 removed by HD. The protocol and all amendments were approved by the Ethics Committee of Huashan Hospital Affiliated to Fudan University (leading site) and the ethics committees of the other study centers.

### Participants

In accordance with the US Food and Drug Administration and China’s National Medicines and Pharmaceutical Administration Guidance for Industry Pharmacokinetics in Patients with Impaired Renal Function, this open-label, phase 1 study enrolled participants with varying degrees of RI, along with a matched healthy control group (14, 15). Eligible participants were Chinese men and women (non-pregnant and non-lactating) between the ages of 18 to 70 years at the time of informed consent, with a body weight of ≥50 kg for men, ≥45 kg for women, and BMI of 18.0 to 32.0 kg/m^2^. All participants were assigned according to their eGFR, calculated using the MDRD formula developed for the Chinese population and standardized by body surface area (BSA): eGFR (mL/min) = [175 × serum creatinine (mg/dL)^−1.234^ × age (years) ^−0.179^ (× 0.739 for women) × BSA]/1.73 (10). BSA was calculated using the DuBois formula (1916): BSA (m^2^) = 0.007184 × Height (cm)^0.725^ × Weight (kg)^0.425^ (16). Participants were classified into the following renal function groups: normal (eGFR, 90–130 mL/min, *n* = 6–8), mild impairment (eGFR, 60–89 mL/min, *n* = 6), moderate impairment (eGFR, 30–59 mL/min, *n* = 6), severe impairment (eGFR, 15–29 mL/min, *n* = 6), and ESRD on HD (eGFR <15 mL/min, *n* = 6). Renal function was expected to be stable, defined as <30% difference in two times eGFR assessed at screening. ESRD participants on HD must be clinically stable with respect to their underlying renal disease and receive intermittent HD at least two to four times per week. Each of six to eight participants with normal renal function (control participants) was matched to the demographic characteristics of RI participants with respect to age (±10 years) and BMI (±20%). The main exclusion criteria included participants with a history of hypersensitivity to cephalosporins or penicillins, a history of kidney transplantation, fluctuating or rapidly deteriorating renal function, or those who required dialysis during the study, except for ESRD participants.

### Treatments

**Fig 4:**
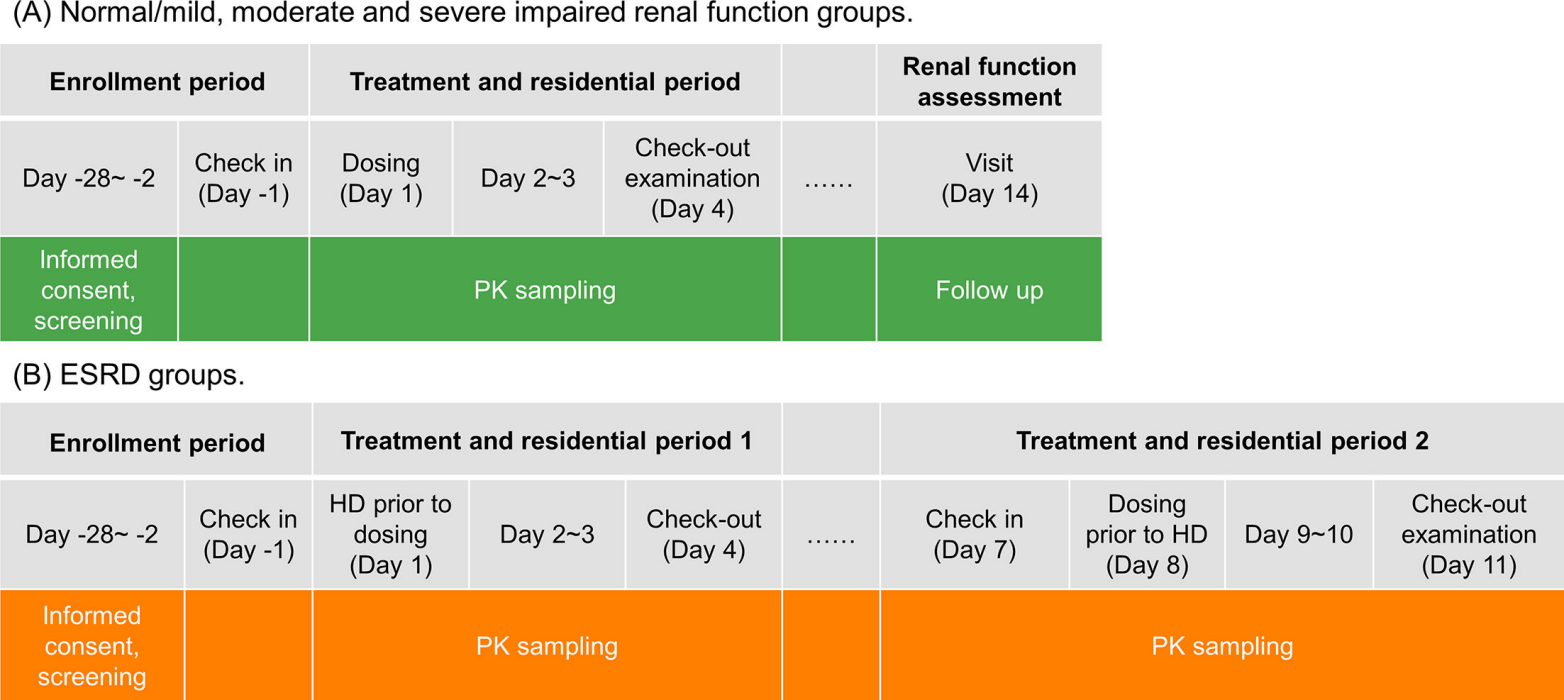
Study design. (**A**) Normal/mild, moderate and severe impaired renal function groups; (**B**) ESRD groups. PK, pharmacokinetics; ESRD, end-stage renal disease; HD, hemodialysis.

The estimated duration of each subject’s participation in this study was approximately two weeks (Fig. 4). The normal renal function and RI participants went through a screening period (not exceeding 28 days), a 1-day baseline period, a 4-day treatment period, and a renal function assessment, which occurred approximately two weeks after the study drug administration. Participants in the normal renal function group and mild-to-severe RI function group received a single intravenous infusion of 1,000 mg HRS-8427 over approximately 1 h. For HD-dependent ESRD participants, HRS-8427 was administered twice: the first dose was administered 2 h after the HD session on day 1 (period 1, without HD), and the second dose was administered 2 h before the HD session on day 8 (after a 7-day washout period, period 2, with HD). Each ESRD participant underwent almost a 4-hour HD session using a high-flux dialyzer. Dialysate flow rate was maintained at 200–300 mL/min, with blood flow rate set at 500 mL/min. The dialyzer membrane surface area ranged from 1.5 to 1.8 m², and identical dialysis equipment was used for both study periods to ensure consistency.

### Sample collection

For quantification of plasma HRS-8427 concentrations, serial blood samples were collected from all cohorts before dosing (0 h) and during infusion session at 0.5, 1 (or at the end of infusion), 2, 3, 4, 6, 8, 12, 24, and 48 h from the start of infusion. Additional blood samples were obtained at 72 h from participants with moderate and severe RI and from ESRD participants. Furthermore, arterial and venous blood samples were collected during the second dialysis session from ESRD participants at 1, 2, 3, and 4 h after dialysis initiation.

Except for the ESRD group, all cohorts were required to provide a spot urine sample collected within 24 h prior to dosing, along with complete urine voids during the following post-dose intervals: 0–8 h, 8–12 h, 12–24 h, 24–36 h, 36–48 h, and 48–72 h. For ESRD participants, spot urine samples (collected −24 to 0 h pre-dose) and complete urine voids during post-dose intervals (0–12 h, 12–24 h, 24–36 h, 36–48 h, and 48–72 h) were required at each dialysis session. The absence of urine production in ESRD participants during predefined time windows was also acceptable with proper documentation.

Dialysate samples were collected at the start of HD (within 10 min) and 1, 2, 3, and 4 h (or at the end of HD) during the dialysis session in period 2.

### Bioanalytical methods

The collected blood samples were immediately placed on ice or refrigerated and centrifuged within 30 min after collection (1,700 × *g*, 10 min, 2 to 8°C). The resultant plasma was stored frozen at −60 to −90°C until analysis. Urine and dialysate samples collected during each collection interval within 60 min were stored frozen at −60°C or below until analysis.

HRS-8427 concentrations in plasma, urine, and dialysate were determined by a validated high‐performance liquid chromatography‐tandem mass spectrometry (Triple Quad 5500, Applied Biosystems/Sciex) assay at bioanalytical laboratory of Nanjing Clinical Tech Laboratories Inc. (China). The assay exhibited linearity from 1 to 600 μg/mL in plasma, 1 to 1,000 μg/mL in urine, and 0.5 to 20 μg/mL in dialysate. Method validation for plasma, urine, and dialysate showed intra-batch and inter-batch precision (coefficient of variation) of <9.5%, <7.8%, and <5.1%, respectively. The accuracy bias of quality control samples ranged from −3.3% to 2.4% in plasma, −0.7% to 1.3% in urine, and −1.6% to 1.2% in dialysate.

### Pharmacokinetic analysis

Mean and standard deviation (SD) for plasma concentrations were calculated by group and sampling time. For descriptive summary analysis, plasma concentrations below the limit of quantification were handled as follows: pre-dose values were set to 0, and post-dose values were set to one-half of the lower limit of quantification. PK parameters for HRS-8427 were calculated using non-compartmental analysis in Phoenix WinNonlin version 8.3 (Certara Inc., Princeton, New Jersey, USA).

The following plasma and urine PK parameters of HRS-8427 were assessed: *C*_max_, time to reach *C*_max_ (*T*_max_), AUC_0–t_, AUC_0_**_–∞_**, *t*_1/2_, CL, and *V*_*z*_. AUC_0–t_ and AUC_0_**_–∞_** values were computed using the linear-up/log-down trapezoidal rule for extrapolation. For each participant with available urinary excretion data, fe and CL_*R*_ were estimated. For participants on HD, Ar and HD clearance (CL_*D*_) were determined.

All statistical analyses were performed using SAS version 9.4 (SAS Institute Inc., Cary, NC, USA). Primary PK parameters of HRS-8427 were log-transformed and analyzed by analysis of variance. Geometric mean ratios (RI vs normal renal function) and 90% CI were calculated.

### Safety assessment

Safety endpoints were monitored from the time of consent through study completion, with follow-up of AEs until resolution, stabilization, or investigator determination of clinical insignificance. The safety and tolerability of HRS-8427 were assessed via monitoring of all AEs, clinical laboratory evaluations (including hematology, blood chemistry tests, and urinalysis), vital signs, electrocardiogram, and physical examinations. AEs were categorized according to the System Organ Class and Preferred Term of Medical Dictionary for Regulatory Activities (version 26.0) and summarized by severity and relationship to the study drug.
